# PREVALENCE AND ASSOCIATED FACTORS FOR POSSIBLE SARCOPENIA IN KOREAN OLDER ADULT FALLERS

**DOI:** 10.1093/geroni/igae098.3411

**Published:** 2024-12-31

**Authors:** Jae Jun Lee, Gwang Suk Kim, Min Kyung Park, Layoung Kim, Namhee Kim, Ji Yeon Lee

**Affiliations:** Yonsei University, Seoul, Republic of Korea; Yonsei University, Seoul, Republic of Korea; Yonsei University College of Nursing, Seoul, Republic of Korea; Yonsei University, Seoul, Republic of Korea; Yonsei University, Seoul, Republic of Korea; Inha University, Incheon, Republic of Korea

## Abstract

Sarcopenia causes adverse health outcomes in older adults, especially in those who have fall experience. Asian Working Group for Sarcopenia (AWGS) introduced possible saropenia for screening sarcopenia in 2019. Identifying the prevalence and associated factors of possible sarcopenia is essential to prevent and manage sarcopenia, however research on possible sarcopenia is still lacking. Therefore, the purpose of this study is to identify the prevalence and associated factors for possible sarcopenia in Korean older adult fallers. This is a cross–sectional study with 184 community-dwelling older adults who aged 65 or over and have at least one fall experience within the last two years. Possible sarcopenia was evaluated based on the AWGS 2019 using calf circumference, five times sit-to-stand test, and handgrip strength. Age, gender, number of fall, depression, cognitive impairment, nutritional status, physical inactivity, and fear of falling were collected and assessed as covariates, and logistic regression analysis was conducted. The mean age of the participants was 74.3±6.5 and 79.3% was female. Among the 184 participants, 47 (25.5%) were possible sarcopenia. Using logistic regression analysis, physical inactivity (odds ratio = 2.55, 95% confidence level = 1.15–5.67) and fear of falling (odds ratio = 1.71, 95% confidence level = 1.01–2.92) were significantly associated with possible sarcopenia, whereas other covariates were not significant. Possible sarcopenia is prevalent in Korean older adult fallers. Our results indicated that encouraging physical activity and making efforts to reduce the fear of falling can be effective strategies for preventing possible sarcopenia in older adult fallers.

